# Transient elevation of cytoplasmic calcium ion concentration at a single cell level precedes morphological changes of epidermal keratinocytes during cornification

**DOI:** 10.1038/s41598-018-24899-7

**Published:** 2018-04-26

**Authors:** Teruasa Murata, Tetsuya Honda, Gyohei Egawa, Yasuo Yamamoto, Ryo Ichijo, Fumiko Toyoshima, Teruki Dainichi, Kenji Kabashima

**Affiliations:** 1 Department of Dermatology, Graduate School of Medicine, Kyoto University, Kyoto, 606-8507 Japan; 2 Central Pharmaceutical Research Institute, Japan Tobacco, Tokyo, Japan; 3 Department of Biosystems Science, Institute for Frontier Life and Medical Science, Kyoto University, Sakyo-ku, Kyoto, 606-8507 Japan; 4 Singapore Immunology Network (SIgN) and Institute of Medical Biology, Agency for Science, Technology and Research (A*STAR), 8A Biomedical Grove, IMMUNOS Building #3-4, Biopolis, 138648 Singapore

## Abstract

Epidermal keratinocytes achieve sequential differentiation from basal to granular layers, and undergo a specific programmed cell death, cornification, to form an indispensable barrier of the body. Although elevation of the cytoplasmic calcium ion concentration ([Ca^2+^]_i_) is one of the factors predicted to regulate cornification, the dynamics of [Ca^2+^]_i_ in epidermal keratinocytes is largely unknown. Here using intravital imaging, we captured the dynamics of [Ca^2+^]_i_ in mouse skin. [Ca^2+^]_i_ was elevated in basal cells on the second time scale in three spatiotemporally distinct patterns. The transient elevation of [Ca^2+^]_i_ also occurred at the most apical granular layer at a single cell level, and lasted for approximately 40 min. The transient elevation of [Ca^2+^]_i_ at the granular layer was followed by cornification, which was completed within 10 min. This study demonstrates the tightly regulated elevation of [Ca^2+^]_i_ preceding the cornification of epidermal keratinocytes, providing possible clues to the mechanisms of cornification.

## Introduction

The body surface of mammalians is covered with skin, which protects them against infection, dehydration, chemicals, and mechanical stress. This barrier function is mainly provided by the stratified squamous epithelium of the skin, the epidermis. The epithelial cells (epidermal keratinocytes) proliferate at the lowest layer of the epidermis and then commence differentiation and upward migration, which takes 40–56 or 8–10 days in humans and mice, respectively^1^. During this differentiation, the morphology of epidermal keratinocytes changes markedly. On the basis of its morphological characteristics, the epidermis can be subdivided into four layers: basal, spinous, granular, and cornified layers. The granular layer is further subdivided into three layers designated as SG1, SG2, and SG3 from the apical to the basal side^2^.

At the border of the granular and cornified layers, epidermal keratinocytes (SG1 cells) undergo a programmed cell death called cornification, which is characterized by a rapid loss of cell volume^3^. Although apoptosis and cornification share similarities, such as the loss of an intact nucleus and other organelles, the dead cell corpses of epidermal keratinocytes are not removed; instead, they are maintained to fulfill a barrier function^4^. The regulatory mechanisms of cornification remain elusive, and their clarification is important to furthering our understanding of skin homeostasis.

The concentration of cytoplasmic calcium ions ([Ca^2+^]_i_) is one of the factors predicted to regulate cornification for several reasons^5,6^. First, it is known that the various forms of cell death (necrosis, apoptosis, and autophagic programmed cell death) share molecular effectors and signaling routes, and the elevation of [Ca^2+^]_i_ is involved in the process of apoptosis, which is assumed to have many similarities to cornification^4,7^. Second, in cultured keratinocytes, [Ca^2+^]_i_ elevation following stimulation for differentiation has been identified^8^. Third, in both the human and murine epidermis, a number of experimental and theoretical analyses have shown the preferential distribution of calcium ions in the granular layer, which are indicated to localize to intracellular compartments^9–14^. Therefore, it has been suspected that the transient elevation of [Ca^2+^]_i_ occurs during the process of differentiation and/or cornification. However, the spatio-temporal dynamics of [Ca^2+^]_i_ in the sequential differentiation of epidermal keratinocytes remains largely unknown because of technical limitations.

In this study, using a two-photon microscope that enables morphological and physiological analyses *in vivo* at subcellular resolution^15–17^, we analyzed the *in vivo* dynamics of [Ca^2+^]_i_ in epidermal keratinocytes. We identified the transient elevation of [Ca^2+^]_i_ in SG1 cells preceding the morphological changes during the process of cornification.

## Results

### [Ca^2+^]_i_ is elevated in epidermal keratinocytes in two layers in a steady state

To visualize [Ca^2+^]_i_, we used mice systemically expressing a genetically encoded Ca^2+^ indicator (GCaMP3)^18^. GCaMP3 is a modified green fluorescent protein (GFP) that increases its fluorescence in proportion to the level of [Ca^2+^]_i_^19^.

**Figure 1:**
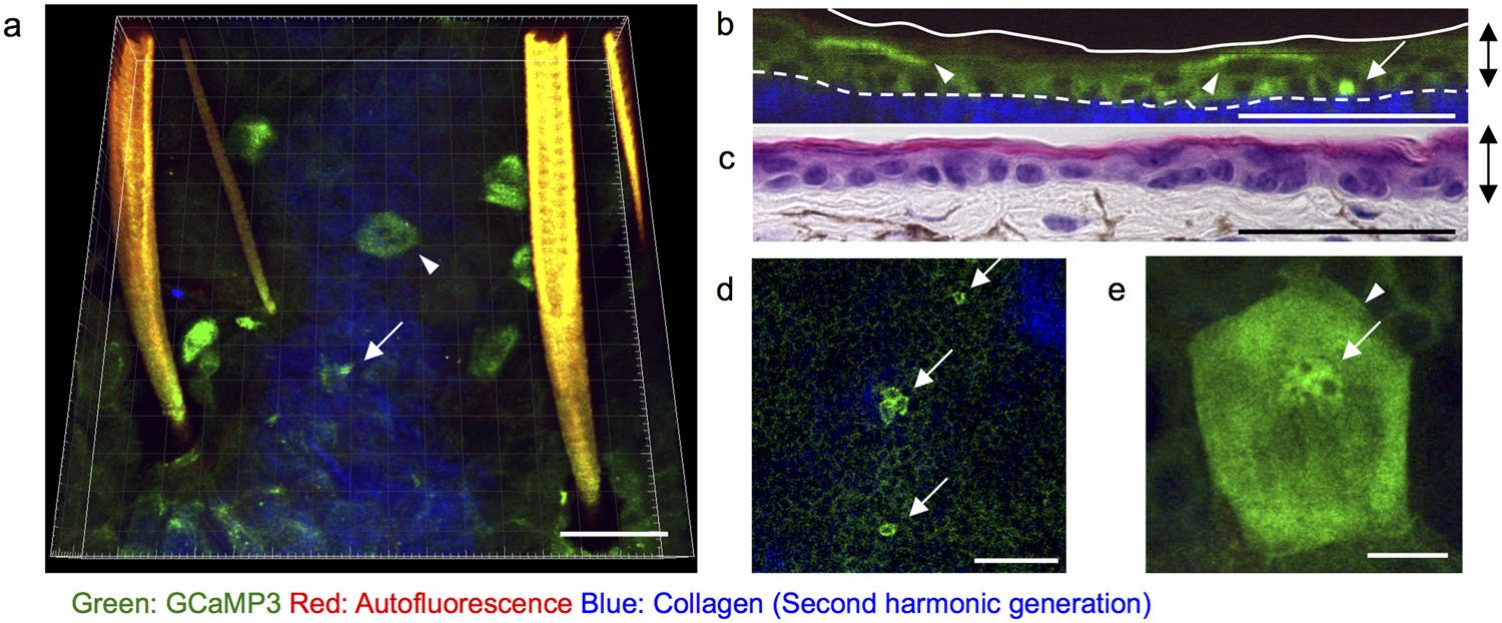
Physiological state of [Ca^2+^]_i_ in the epidermis. Representative two-photon images of the ear skin of mice systemically expressing GCaMP3 (GCaMP3 mice). (**a**) Three-dimensional view, (**b**) Vertical view showing cells with elevated fluorescence of GCaMP3 (GCaMP3^high^ cells). Arrow and arrowhead: GCaMP3^high^ cells in the basal and granular layers, respectively. Green: GCaMP3; red: autofluorescence; blue: second-harmonic generation from dermal collagen. Dashed line in (b): dermo-epidermal junction. (**c**) Hematoxylin and eosin staining of the ear skin section. Double-headed arrows: epithelial layers. (**d**) Horizontal view of the basal layer. Arrows: GCaMP3^high^ basal cells. (**e**) Maximum intensity view of the granular layer. Arrowhead: a GCaMP3^high^ granular cell. Arrow: nucleus. Scale bars: 50 μm (a–d) and 10 μm (**e**). [Ca^2+^]_i_: concentration of calcium ions in the cytoplasm. Results are representative of at least three independent experiments.

In ear skin in a steady state, cells with an increased fluorescence of GCaMP3 (GCaMP3^high^ cells) were identified in a subset of epidermal keratinocytes in two layers: the lowest layer of the epidermis (basal layer) and the upper epidermis (granular layer) (Fig. 1a–c). The elevated fluorescence of GCaMP3 was detected in the cytoplasm in the basal layer (Fig. 1d), whereas it was detected in both the nucleus and the cytoplasm in the granular layer (Fig. 1e).

To dissect whether this difference in the localization of elevated GCaMP3 fluorescence between basal and granular cells actually reflects the difference of [Ca^2+^]_i_ elevation or derives from uneven subcellular distribution of GCaMP3 protein, we immunostained GCaMP3 protein with anti-GFP antibody using sections of fingertip skin of GCaMP3 mice. In the basal cells, the protein of GCaMP3 distributed preferentially in the cytoplasm, whereas it was hardly detected in the nucleus (Supplemental Fig. 1). In contrast, in the granular cells, the protein of GCaMP3 was detected in both the cytoplasm and nucleus (Supplemental Fig. 1). Therefore, the lack of elevated fluorescence of GCaMP3 in the nucleus of basal cells might be due to the low concentration of the GCaMP3 protein.

### A Ca^2+^ spike in the basal layer occurs on the second time scale in three distinct patterns

**Figure 2:**
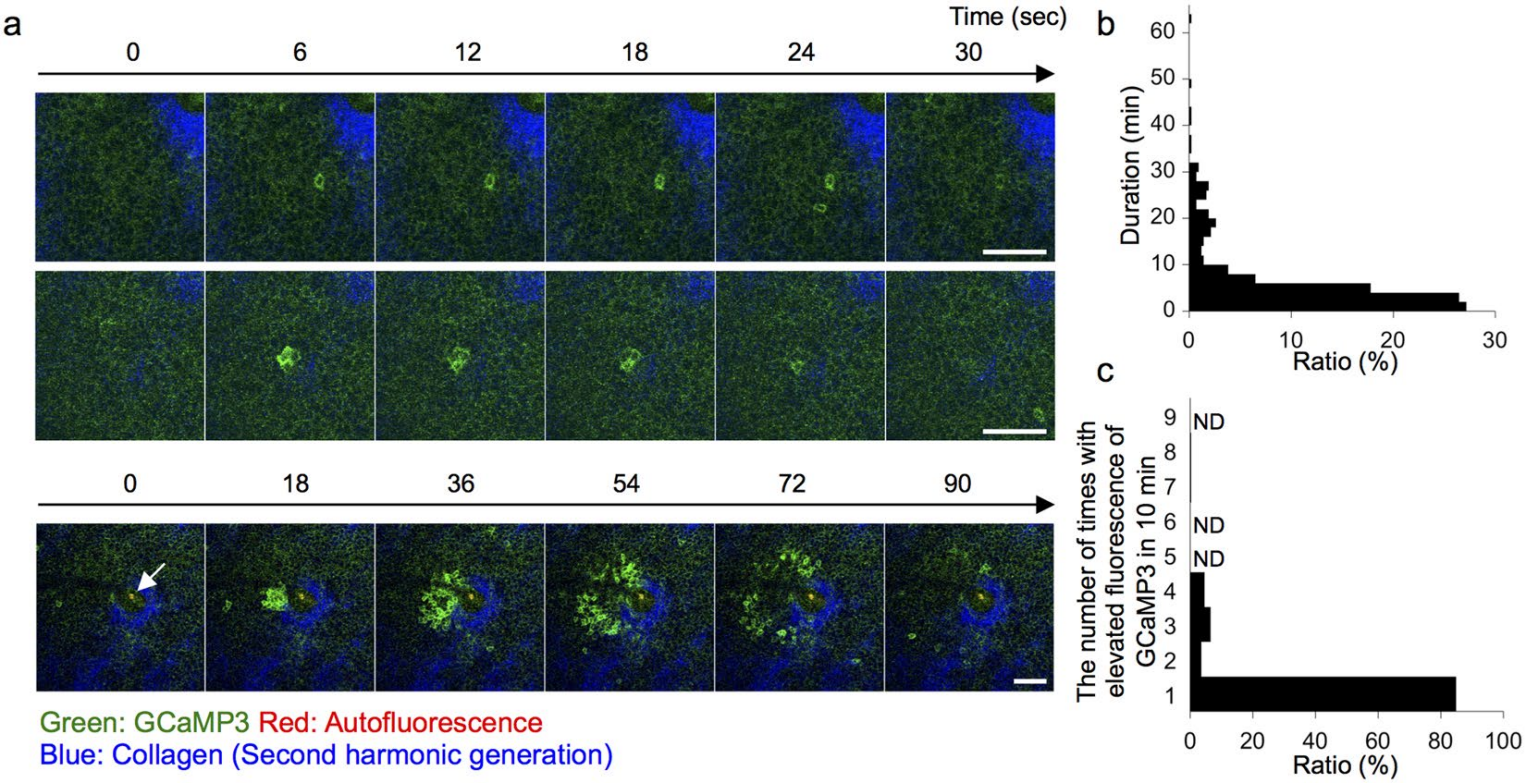
Three patterns of transient [Ca^2+^]_i_ elevation in the basal layer. (**a**) Representative time-lapse images of the basal layer of the ear skin of GCaMP3 mice. Upper panel: Ca^2+^ spike in a single cell unit. Middle panel: clustered Ca^2+^ spike. Lower panel: radially spreading Ca^2+^ spike. Arrow: hair shaft. Green: GCaMP3; red: autofluorescence; blue: second-harmonic generation from dermal collagen. (**b**) Relationship between the duration and the proportion of Ca^2+^ spikes in a single cell unit (n = 416 events). (**c**) Relationship between the number of times and the proportion of Ca^2+^ spikes in a single cell unit (n = 310 cells) in 10 min. ND: not detected. Scale bars: 50 μm. Results are representative of at least three independent experiments.

Next, we evaluated temporal changes in the basal layer of the ear skin. The appearance of GCaMP3^high^ cells had three spatiotemporally different patterns (Fig. 2a–c).

The most common pattern was a Ca^2+^ spike in a single cell unit. The fluorescence of GCaMP3 elevated sporadically as single cells (Fig. 2a, upper panel, and Supplemental Movie 1). This occurred at both follicular and intrafollicular epithelia, and lasted for approximately 4 s (median value, Fig. 2b). Although most of the basal cells exhibited a Ca^2+^ spike only once during the observation period, some of the cells exhibited a Ca^2+^ spike several times in 10 min (Fig. 2c).

The second pattern was a clustered Ca^2+^ spike. GCaMP3^high^ cells appeared sporadically as clustered cells less frequently than a Ca^2+^ spike in a single cell unit (Fig. 2a, middle panel). The duration of this pattern was also on the second time scale (Supplemental Movie 2). A clustered Ca^2+^ spike occurred around an oval GCaMP3^high^ cell that was larger than basal keratinocytes, although this observation was quite rare (Supplemental Movie 3). The frequency of both a Ca^2+^ spike in a single cell unit and a clustered Ca^2+^ spike pattern was much lower in the hind paw skin (Supplemental Movie 4) than in the ear skin, suggesting that the fundamental level of [Ca^2+^]_i_ in basal keratinocytes might differ significantly depending on the skin site.

The third pattern was a radially spreading Ca^2+^ spike. The elevation of the fluorescence of GCaMP3 sometimes originated from a few cells and radially propagated in the basal layer of the epidermis (Fig. 2a, lower panel, and Supplemental Movie 5). The mean duration and the radius of this pattern were 89.4 ± 5.5 s and 86.7 ± 6.9 μm (n = 19 events), respectively. The speed of radial propagation was 0.95 ± 0.04 μm/s or approximately 6.1 basal cells/min (n = 19 events). This pattern occurred several times at intervals of 139.8 ± 13.3 s (n = 10 sequential events), and the origin was always located around the hair follicles (Fig. 2a, lower panel). A radially spreading Ca^2+^ spike was not observed in hind paw skin that lacks hair follicles (Supplemental Movie 4).

The frequency of these three patterns of Ca^2+^ spikes did not linearly correlate with the strength of the excitation laser (Supplemental Fig. 2), indicating that these were not dependent on artifacts of laser stimulation but were physiological phenomena in a steady state.

### [Ca^2+^]_i_ in the granular layer is elevated transiently for approximately 40 min

Next, we addressed the dynamics of elevation of [Ca^2+^]_i_ in the granular layer.

**Figure 3:**
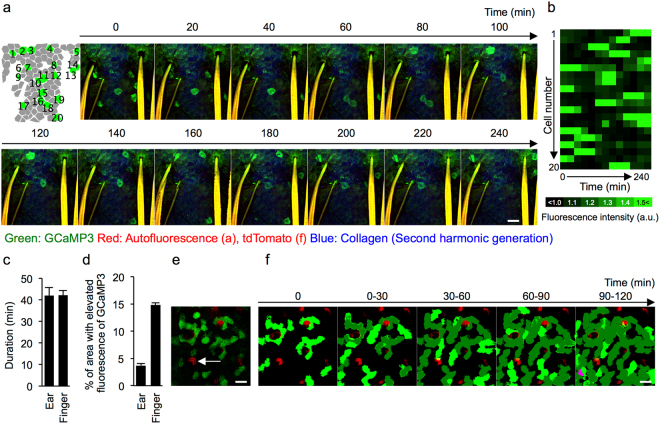
Dynamics of [Ca^2+^]_i_ elevation in the granular layer. (**a**) Schematic (far left) and representative time-lapse images of the granular layer of the ear skin of GCaMP3 mice. Green: GCaMP3; red: autofluorescence; blue: second-harmonic generation from dermal collagen. (**b**) Heat map showing the relative intensity of the fluorescence of GCaMP3 in each granular cell. Note that each cell exhibits a transient elevation of fluorescence only once in the observation period. (**c**) Duration of transient [Ca^2+^]_i_ elevation in the granular cells in ear skin (n = 10 cells) and fingertip skin (n = 17 cells). (**d**) Percentage of area with elevated fluorescence of GCaMP3 in an observation field in the ear and fingertip skin. Overall, 13 and 25 time points were analyzed in the ear and fingertip skin, respectively. (**e**) Representative two-photon image of the fingertip skin of mice systemically expressing GCaMP3 and tandem dimer Tomato (GCaMP3/tdTomato mice). Arrow: intra-epidermal sweat duct. Green: GCaMP3, red: tdTomato. (**f**) Time-projection image showing the order of transient [Ca^2+^]_i_ elevation in the fingertip skin. Bright green: newly appearing cells with transient [Ca^2+^]_i_ elevation; dark green: areas that showed transient [Ca^2+^]_i_ elevation previously; magenta: area that showed overlap of transient [Ca^2+^]_i_ elevation. Error bars: standard error of the mean. Scale bars: 50 μm. Results are representative of at least three independent experiments.

Time-lapse imaging showed that the elevation of the fluorescence of GCaMP3 was transient (Fig. 3a,b, Supplemental Movie 6). The mean duration of elevated fluorescence was approximately 40 min in both ear and fingertip skin (Fig. 3c). The frequency of GCaMP3^high^ cells was much higher in the fingertip skin than in the ear skin (Fig. 3d). The estimated times within which the elevation of [Ca^2+^]_i_ took place in the entire observational area were 23.4 h and 4.7 h in the ear and fingertip skin, respectively. GCaMP3^high^ cells in the granular layer appeared at a single cell level and did not spread to adjacent cells (Fig. 3a). Furthermore, GCaMP3^high^ cells rarely overlapped at the same horizontal position within the observational period (Fig. 3e,f).

Taken together, these results suggest that the order and the duration of [Ca^2+^]_i_ elevation were tightly regulated in each granular cell that eventually achieved cornification.

### [Ca^2+^]_i_ is elevated in a subset of SG1 cells

Next, we investigated the precise location of GCaMP3^high^ cells in the granular layer. The granular layer is subdivided into three layers, and granular cells in the middle layer (SG2 cells) form a tight junction barrier^2,20^. Subsequently, granular cells in the most apical layer (SG1 cells) finally achieve cornification, and then become integrated into the cornified layer.

**Figure 4:**
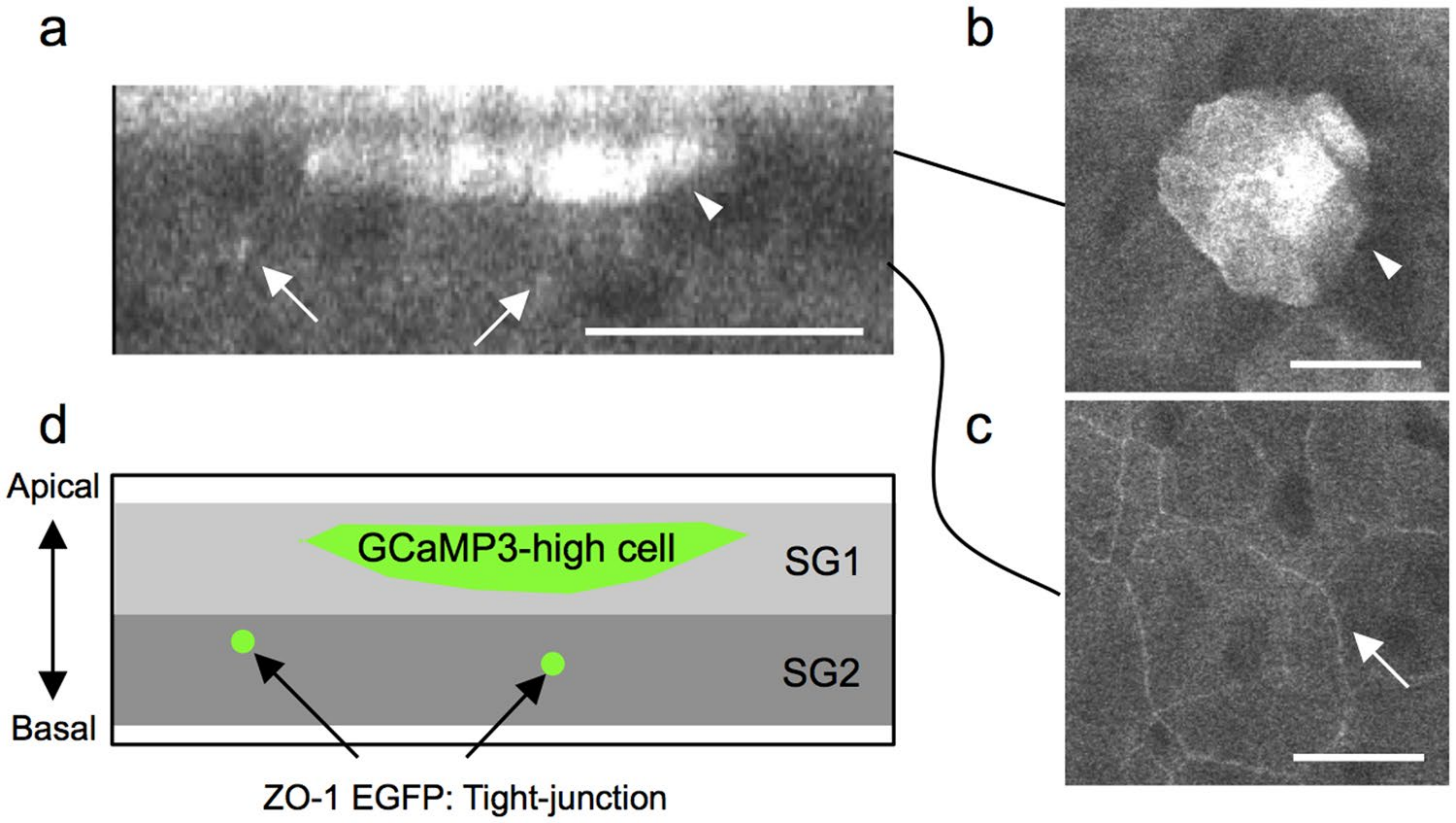
Location of GCaMP3^high^ cells and tight junctions in the granular layer. (**a-c**) Representative two-photon images of the hind paw skin of mice systemically expressing both GCaMP3 and ZO-1 EGFP. The intensity of green fluorescence from both GCaMP3 and ZO-1 EGFP is represented in gray scale. (**a**) Vertical view. (**b,c**) Horizontal view. Arrows: fluorescence from ZO-1 EGFP that corresponds to tight-junctions. Arrowheads: a granular cell showing transient [Ca^2+^]_i_ elevation. (**d**) Schematic of the location of [Ca^2+^]_i_-high granular cells and tight junctions. SG1 and SG2: The most apical and middle layers of the granular layer, respectively. Scale bars: 20 μm.

To visualize the SG2 layer, we utilized mice systemically expressing ZO-1 EGFP, and crossbred them with GCaMP3 mice^21^ (ZO-1 EGFP/GCaMP3 mice). In ZO-1 EGFP mice, zonula occludens-1 (ZO-1), a constituent molecule of tight junctions, is fused with enhanced green fluorescent protein (eGFP). Using these mice, we can visualize the SG2 layer by exciting eGFP. In the ear and hind paw skin of ZO-1 EGFP/GCaMP3 mice, GCaMP3^high^ cells were located above the tight-junction signal (Fig. 4)^20^, indicating that the transient elevation of [Ca^2+^]_i_ occurs in SG1 cells.

**Figure 5:**
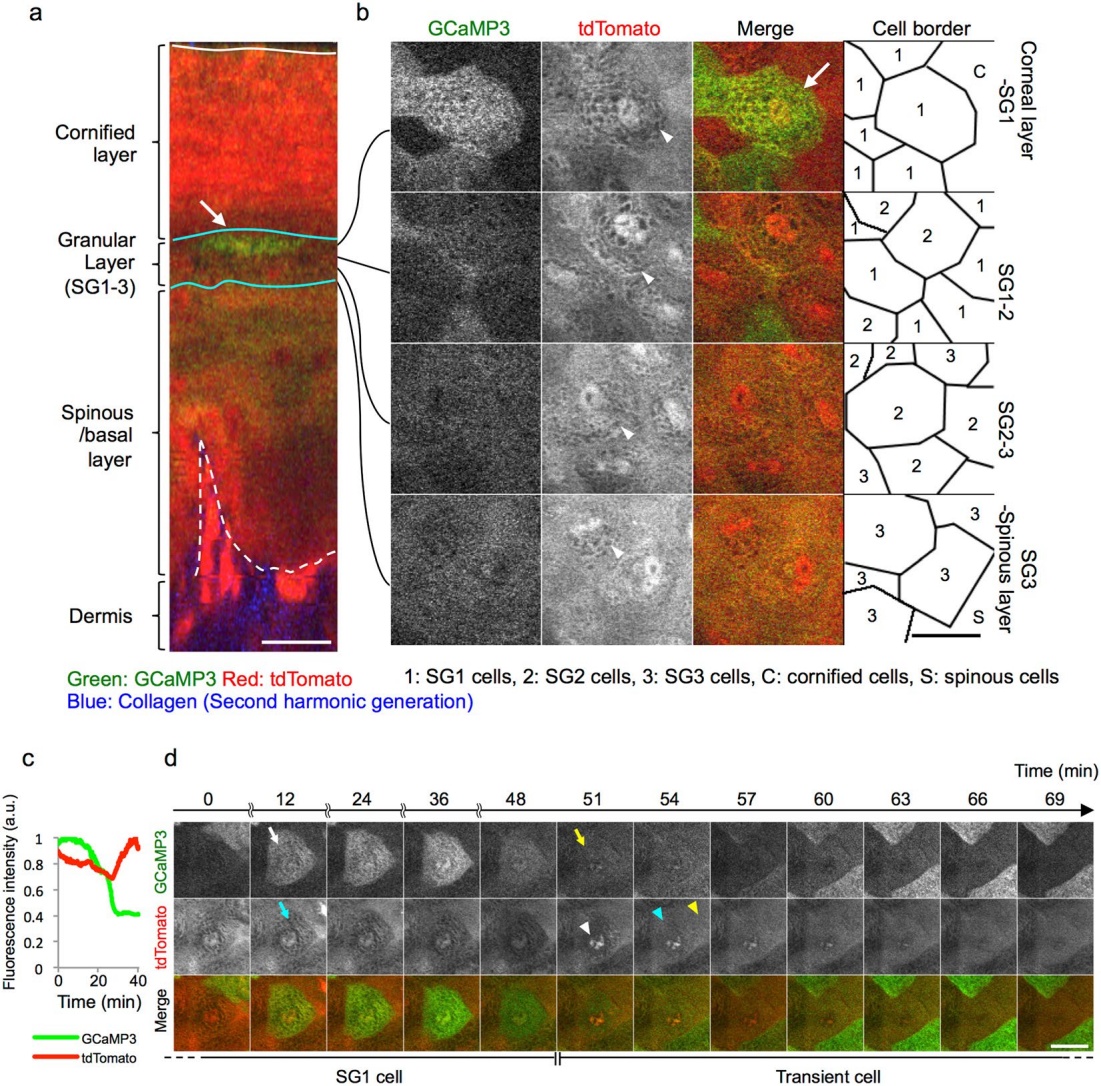
Temporal relationship between transient [Ca^2+^]_i_ elevation and cornification. (**a,b**) Representative images of two-photon microscope of the fingertip skin of GCaMP3/tdTomato mice. **(a)** Vertical view. Note that cornified cells exhibit intensified fluorescence of tdTomato. Arrows: granular cells with transient [Ca^2+^]_i_ elevation. White line: the surface of the skin. Cyan lines: upper and lower borders of the granular layer. Dashed white line: dermo-epidermal junction. (**b**) Horizontal view showing the granular layer at different depths. Note that nuclei and cytoplasmic granules are visualized with the fluorescence of tdTomato. (**c**) Representative plot of the relative fluorescence intensity of GCaMP3 (green line) and tdTomato (red line) in the process of transient [Ca^2+^]_i_ elevation and cornification. (**d**) Time-lapse imaging of the most apical granular cell. A white arrow indicates the start of [Ca^2+^]_i_ elevation, and a yellow arrow indicates the end of [Ca^2+^]_i_ elevation. Cyan arrow: cytoplasmic granules. White arrowhead: abrupt elevation of the fluorescence of tdTomato in the nucleus. Cyan arrowhead: disappearance of cytoplasmic granules. Yellow arrowhead: cornified cell that becomes indistinguishable from the neighboring cell. Green: GCaMP3, red: tdTomato, blue: second-harmonic generation from dermal collagen. Scale bars: 20 μm. Results are representative of at least three independent experiments.

To further confirm this observation, we next used tandem dimer Tomato (tdTomato) mice. Tdtomato is one of the red fluorescent proteins, which was specifically designed for low aggregation^22^. In this strain, the morphology of epidermal keratinocytes (the nuclei and intracellular granules in the granular layer) was well visualized with the red fluorescence of tdTomato. In addition, the border of the granular and cornified layers was visualized by contrast with fluorescence intensity (Fig. 5a), which may have been be due to the condensation of fluorescent protein arising from the reduction of cell volume during cornification. We crossbred tdTomato mice with GCaMP3 mice (tdTomato/GCaMP3 mice) and observed their ear and hind paw skin. Consistent with the results in ZO-1 EGFP/GCaMP3 mice, GCaMP3^high^ cells were located immediately beneath the cornified layer (Fig. 5a). During the transient elevation of the fluorescence of GCaMP3, these cells sustained the typical morphology of granular cells: intact nuclear structure and intracellular granules (Fig. 5c).

Taken together, these results demonstrate that a subset of SG1 cells residing immediately beneath the cornified layer showed the transient elevation of [Ca^2+^]_i_.

### Morphological changes of keratinocytes from the granular layer to the cornified layer begin seconds after the transient elevation of [Ca^2+^]_i_

Next, we investigated the association between the transient elevation of [Ca^2+^]_i_ and the transition from granular to cornified cells using tdTomato/GCaMP3 mice.

Seconds after the end of the decrease in the fluorescence of GCaMP3, the fluorescence intensity of tdTomato began to increase (Fig. 5c). Then, within 3 min of the decrease in the fluorescence of GCaMP3, the nucleus exhibited transformation, followed by the disappearance of intracellular granules (Fig. 5d, Supplemental Movie 7). Subsequently, the nuclear and cytoplasmic signals of tdTomato became homogeneous, and these cells became indistinguishable from adjacent cornified cells (Fig. 5d, Supplemental Movie 7), which occurred within 10 min in both ear and fingertip skin. Morphological changes and the transient elevation of the fluorescence of GCaMP3 were paired in all cells examined (Table 1).

**Table 1:** Evaluation of relationships of transient [Ca^2+^]_i_ elevation and morphological changes associated with cornification.

Automatically detected cells with	Manual evaluation of	
[Ca^2+^]_i_ increase (40 cells)	Cornification	
	+	−
	40 (100%)	0 (0%)
Cornification (64 cells)	[Ca^2+^]_i_ increase	
	+	−
	64 (100%)	0 (0%)

**Figure 6:**
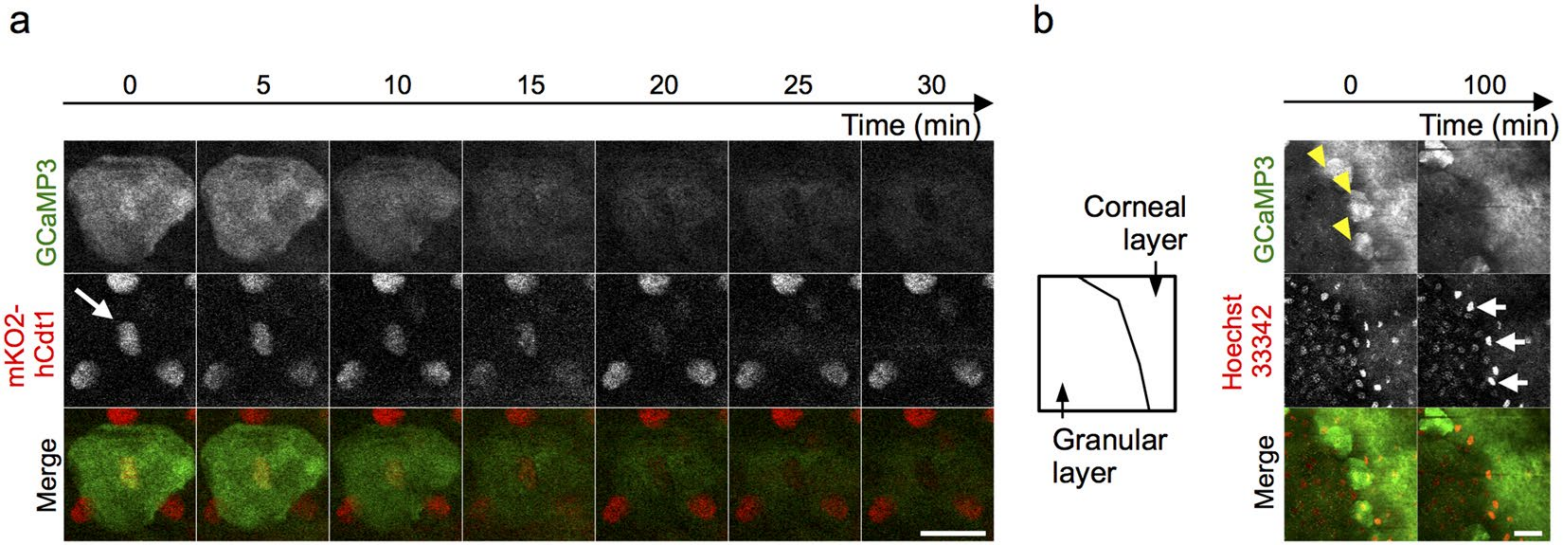
Temporal relationship between the transient [Ca^2+^]_i_ elevation and changes in the nucleus. (**a**) Time-lapse imaging of the most apical granular cell in the hind paw of mice systemically expressing Fucci and GCaMP3. Arrow: nuclear signal of red fluorescent protein of Fucci system. Note that the nuclear signal disappeared along with the decrease in the fluorescence of GCaMP3. (**b**) Time-lapse imaging of the granular and cornified layers of the hind paw skin of GCaMP3 mice injected with Hoechst 33342. Yellow arrowheads: granular cells with elevated fluorescence of GCaMP3. White arrows: intensified fluorescence of Hoechst 33342. Green: GCaMP3; red: mKO2-hCdt1 (a), Hoechst 33342 (b). Scale bars: 20 μm. Results are representative of at least two independent experiments.

To further characterize the nuclear changes in the transition from granular to cornified cells, we used Fucci mice and crossbred them with GCaMP3 mice (Fucci/GCaMP3 mice). In Fucci mice, the nucleus is labeled with green and red fluorescent protein, and the ratio of these proteins indicates the cell cycle. The nuclear signal disappeared immediately after the decrease in the fluorescence of GCaMP3 (Fig. 6a), indicating that some cell death other than apoptosis had occurred, because apoptotic cells exhibit fragmented nuclei but do not lose the fluorescent label^23^.

We next assessed temporal changes in the DNA of the nucleus by labeling it with Hoechst 33342^24,25^. The fluorescence of Hoechst 33342 gradually increased over 100 min after the decrease in GCaMP3 fluorescence (Fig. 6b) and then disappeared (Supplemental Fig. 2). These results suggest that the condensation of DNA, possibly because of the reduction in cell volume by cornification, occurred after the decrease of [Ca^2+^]_i_, which is followed by the degradation of DNA in the process of cell death (Supplemental Fig. 2).

Taken together, these results indicate that the transition from SG1 cells to cornified cells occurs within a short time after the transient elevation of [Ca^2+^]_i_.

### [Ca^2+^]_i_-high cells are observed in the upper layer of various types of cornifying stratified epithelia

To evaluate whether the transient elevation of [Ca^2+^]_i_ is a common feature of epithelia that undergo cornification, we carried out the *in vivo* and *ex vivo* observations of various stratified squamous epithelia. Stratified squamous epithelia with cornified layers are classified into two types: cornification with enucleation (orthokeratosis) and cornification without enucleation (parakeratosis). Most parts of the skin exhibit orthokeratosis, whereas some parts of the skin and most of the mucous epithelia with cornified layers exhibit parakeratosis.

**Figure 7:**
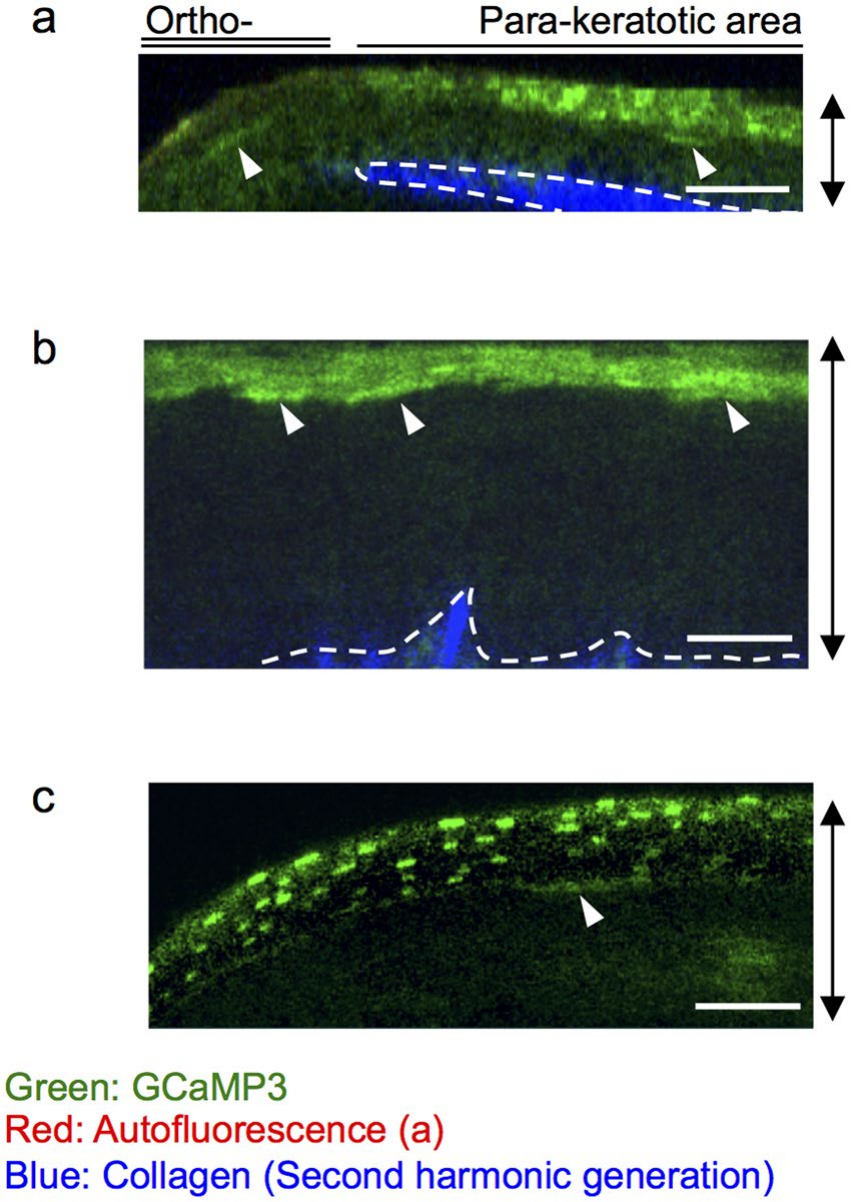
Physiological state of [Ca^2+^]_i_ in the parakeratotic epidermis and mucous epithelia. Representative two-photon images of GCaMP3 mice (a,b) or GCaMP3/tdTomato (c), in a vertical view. (**a**) *In vivo* observation of the tail skin. (**b,c**) *Ex vivo* observation of the mucous epithelia of the (**b**) vagina and (**c**) oral cavity. The contrast in the fluorescence of GCaMP3 was corrected using the fluorescence signal of tdTomato, which reflects the concentration of fluorescent proteins in the cytoplasm. Arrowheads: cells with elevated fluorescence of GCaMP3 beneath the cornified layer. Dashed lines: dermo-epidermal junction or lower edge of the mucous epithelia. Green: GCaMP3; red: autofluorescence (a); blue: second-harmonic generation from dermal collagen. Double-headed arrows: epithelial layers. Scale bars: 50 μm. Results are representative of at least three independent experiments.

We examined the tail skin *in vivo*; at this site, both orthokeratosis and parakeratosis coexist under physiological conditions^26^. Elevated fluorescence of GCaMP3 was observed in the cytoplasm and the nucleus in a subset of SG1 cells in both orthokeratotic and parakeratotic areas (Fig. 7a). In addition, in the parakeratotic area, residual nuclei in the cornified layer showed intense fluorescence of GCaMP3 (Fig. 7a). Next, we examined the parakeratotic area of the oral mucosa (hard palate) or vagina by *ex vivo* observation and identified GCaMP3^high^ cells beneath the cornified layer (Fig. 7b,c). These results suggest that the transient elevation of [Ca^2+^]_i_ in SG1 cells is general in cornifying epithelia, regardless of enucleation.

## Discussion

Here, we elucidated the physiological dynamics of [Ca^2+^]_i_ in epidermal keratinocytes. We identified three patterns of Ca^2+^ spikes in the basal layer, and the transient elevation of [Ca^2+^]_i_ in SG1 cells preceding the morphological changes of cornification.

To the best of our knowledge, spontaneous Ca^2+^ spikes in the basal layer of the epidermis have never been documented. A Ca^2+^ spike in a single cell unit might be a feature shared among epithelial cells because a similar phenomenon was observed in the intestinal epithelium^27^. A clustered Ca^2+^ spike was very occasionally observed to occur frequently in the vicinity of uncharacterized large oval GCaMP3^high^ cells. This suggests that a clustered Ca^2+^ spike might reflect the stimulation of basal keratinocytes by dermal cells. A radially spreading Ca^2+^ spike might be related to hair follicles. In addition, this pattern might be transmitted via gap junctions, because Ca^2+^ propagation through gap junctions has been detected in the basal layer of *ex vivo* human skin^28^. Importantly, all patterns of Ca^2+^ spikes were sporadic and occurred several times in the same cells. Furthermore, it was shown that the level of [Ca^2+^]_i_ oscillation regulates the activity of stem cells in the intestine of *Drosophila*^29^. Therefore, we speculated that these events might be correlated with the proliferation or early differentiation of epidermal keratinocytes rather than cornification.

Our study revealed that the transient elevation of [Ca^2+^]_i_ occurred at a single cell level in SG1 cells, which has not been identified by previous studies showing a high concentration of calcium ions in the granular layer^9–11^. This result fits with the recent *in vivo* studies showing the terminal differentiation of keratinocytes in an apparently sporadic manner^2,30^. Because the transient elevation of [Ca^2+^]_i_ rarely occurred at the same horizontal position within the observational period, the order of this event is unlikely to be completely stochastic; rather, it is probably regulated by some unknown mechanisms. The frequency of the elevation of [Ca^2+^]_i_ in SG1 cells might reflect the speed of turnover of the epidermal keratinocytes, which depend on the site of skin^31^.

The transient elevation of [Ca^2+^]_i_ in SG1 cells was coupled with and occurred just before the morphological changes associated with cornification. Although the causal relationship between the elevation of [Ca^2+^]_i_ and cornification was not evaluated in this study, our data strongly suggest that the transient elevation of [Ca^2+^]_i_ is involved in the mechanisms of cornification. Indeed, previous studies demonstrated rapid increase in the number of transitional cells after elevation of [Ca^2+^]_i_ by an inhibition of sarco/endoplasmic reticulum Ca^2+^ ATPase (SERCA)^12,32^. Furthermore, it is known that Ca^2+^ is required to activate the transglutaminases that assemble the cornified envelope, a protective barrier structure inside the plasma membrane^33^. In addition, Ca^2+^ influx induces the formation of the cornified envelope in cultured keratinocytes^34,35^. Therefore, it is possible that elevated [Ca^2+^]_i_ activates transglutaminases in SG1 cells before the transformation to cornified cells.

GCaMP3-protein was distributed exclusively in the cytoplasm in the basal cells, but distributed to both the nucleus and cytoplasm in the granular cells. This observation raises a hypothesis that GCaMP3 protein in the cytosol re-distributed to nucleus due to increased permeability of nuclear envelope in the process of cell death^36^. In this scenario, the initiation of cell death precedes the transient elevation of [Ca^2+^]_i_. Because the elevated fluorescence of GCaMP3 disappears in skin sections for immunohistochemistry, establishment of an *in vivo* evaluation system to detect GCaMP3 fluorescence and keratinocyte differentiation markers or cell death markers would be necessary to clarify this issue.

Gene mutations related to Ca^2+^ homeostasis result in skin lesions, including aberrantly promoted cornification in congenital diseases such as keratitis-ichthyosis-deafness (KID) syndrome and Darier’s disease^37,38^. This implies a possible link between Ca^2+^ homeostasis and cornification. In KID syndrome, mutated connexin proteins were recently shown to form hyperactive hemi channels, connecting the inside and outside of the cells^39,40^. Therefore, Ca^2+^ influx through hyperactive hemi channels in the granular layer might promote cornification in the skin of patients with KID syndrome. Whereas, Darier’s disease is caused by a mutation in *ATP2A2* coding type 2 isoform of SERCA (SERCA2)^38^. Because calcium ions in the granular layer are suggested to localize to intracellular compartments including endoplasmic reticulum (ER), it is possible that the source of calcium ions in the transient elevation of [Ca^2+^]_i_ in SG1 cells are intracellular compartments^13,14^. Generally, the high concentration of calcium ions in these compartments is sustained by calcium pumps or channels including SERCA2. Therefore, mutated SERCA2 proteins might affect the concentration of calcium ions in ER and the dynamics of [Ca^2+^]_i_ in SG1 cells, eventually resulting in aberrant cornification in the granular layer in Darier’s disease.

In conclusion, we identified the transient elevation of [Ca^2+^]_i_ in SG1 cells preceding cornification. This finding significantly deepens our understanding of the mechanisms of cornification in epidermal keratinocytes and may lead to the development of specific treatment for skin diseases affecting the cornified layer.

## Materials and Methods

### Mice

R26-CAG-LoxP-GCaMP3 mice^18^ (RBRC05163; RIKEN BRC, Ibaraki, Japan) and R26-CAG-tdTomato mice (007908; Jackson Laboratory, Bar Harbor, Maine, USA) were crossbred with EIIa-cre mice (Jackson Laboratory) to generate mice systemically expressing fluorescent proteins. These mice were further crossbred with R26-ZO1-EGFP^21^ or R26p-Fucci2^41^ mice (CDB0256K and CDB0203T; RIKEN CLST[http://www2.clst.riken.jp/arg/reporter_mice.html], Kobe, Japan). Both male and female mice were applied for experiments at their age of 8–24 weeks. All experimental protocols were approved by the Animal Experimentation Committee of Kyoto University and all animal experimental procedures were performed according to the Animal Protection Guidelines of Kyoto University.

### Two-photon microscope

*In vivo* two-photon imaging of the skin was performed as previously described^16^. In brief, mice were anesthetized with isoflurane and positioned on the heating plate on the stage of a two-photon microscope (IX-81; Olympus, Tokyo, Japan). The earlobes, feet, and tails were fixed with a single drop of immersion oil. We checked the correlations between the intensity of excitation laser and the frequency of Ca^2+^ spikes in the basal layer (Supplemental Fig. 2), and determined the observation settings that do not evoke artifacts by heat or laser stimulation (Supplemental Table). In certain experiments, Hoechst 33342 (Invitrogen, Carlsbad, CA, USA) was injected into the hind paw at a concentration of 1 mg/mL, 1 day before observation. After confirming that the fluorescence pattern of GCaMP3 at the granular layer did not change within 5 min after skin excision, we performed *ex vivo* imaging of the mucous epithelia within 5 min after the excision of each organ.

### Immunostaining

Freshly excised fingertip skin was immersed with 4% paraformaldehyde, 20% sucrose overnight, respectively. Samples were then embedded in the OCT compound (Sakura, Tokyo, Japan) and was frozen in liquid nitrogen. Sections of 5 μm thickness were stained with anti-GFP antibody (A11122; Molecular Probes, Carlsbad, CA, USA) and anti-rabbit IgG antibody (A21207; Invitrogen, Carlsbad, CA, USA). Fluorescence images were obtained using a fluorescence microscope (BZ-9000; Keyence, Osaka, Japan).

### Image analysis

Images were processed using ImageJ version 1.45 s (NIH, Bethesda, USA) and Imaris software version 7.2.1 (Bitplane, Zurich, Switzerland). The elevation of the fluorescence of GCaMP3 was determined in time series images, with a threshold of a 50% increase relative to the mean fluorescence intensity of each cell.

## Electronic supplementary material

Supplemental Information

Supplemental Movie 1: Ca2+ spike in a single cell unit in the basal layer

Supplemental Movie 2: Clustered Ca2+ spike in the basal layer

Supplemental Movie 3: Clustered Ca2+ spike frequently observed around a continuously fluorescent cell in the basal layer

Supplemental Movie 4: Ca2+ spikes in the basal layer of hind paw skin

Supplemental Movie 5: Radially spreading Ca2+ spike in the basal layer

Supplemental Movie 6: Transient [Ca2+]i elevation in the granular layer

Supplemental Movie 7: Cornification of granular cells following the transient [Ca2+]i elevation
