# Supplementary material for: Transient elevation of cytoplasmic calcium ion concentration at a single cell level precedes morphological changes of epidermal keratinocytes during cornification
Source: Sci Rep. 2018 Apr 26;8:6610. doi: 10.1038/s41598-018-24899-7 (PMC5919969; doi:10.1038/s41598-018-24899-7)
Supplement: Supplementary file 1 — Supplemental Information [file 41598_2018_24899_MOESM1_ESM.pdf]

Transient elevation of cytoplasmic calcium ion concentration at a single cell level precedes morphological changes of epidermal keratinocytes during cornification

Teruasa Murata[1], Tetsuya Honda*[1], Gyohei Egawa[1], Yasuo Yamamoto[1,2], Ryo Ichijo[3], Fumiko Toyoshima[3], Teruki Dainichi[1], and Kenji Kabashima*[1,4,]

[1]Department of Dermatology, Graduate School of Medicine, Kyoto University, Kyoto 606-8507, Japan

[2]Central Pharmaceutical Research Institute, Japan Tobacco, Japan

[3]Department of Biosystems Science, Institute for Frontier Life and Medical Science, Kyoto University, Sakyo-ku, Kyoto 606-8507, Japan

[4]Singapore Immunology Network (SIgN) and Institute of Medical Biology, Agency for Science, Technology and Research (A*STAR), 8A Biomedical Grove, IMMUNOS Building #3-4, Biopolis 138648, Singapore

Supplemental Figure 1

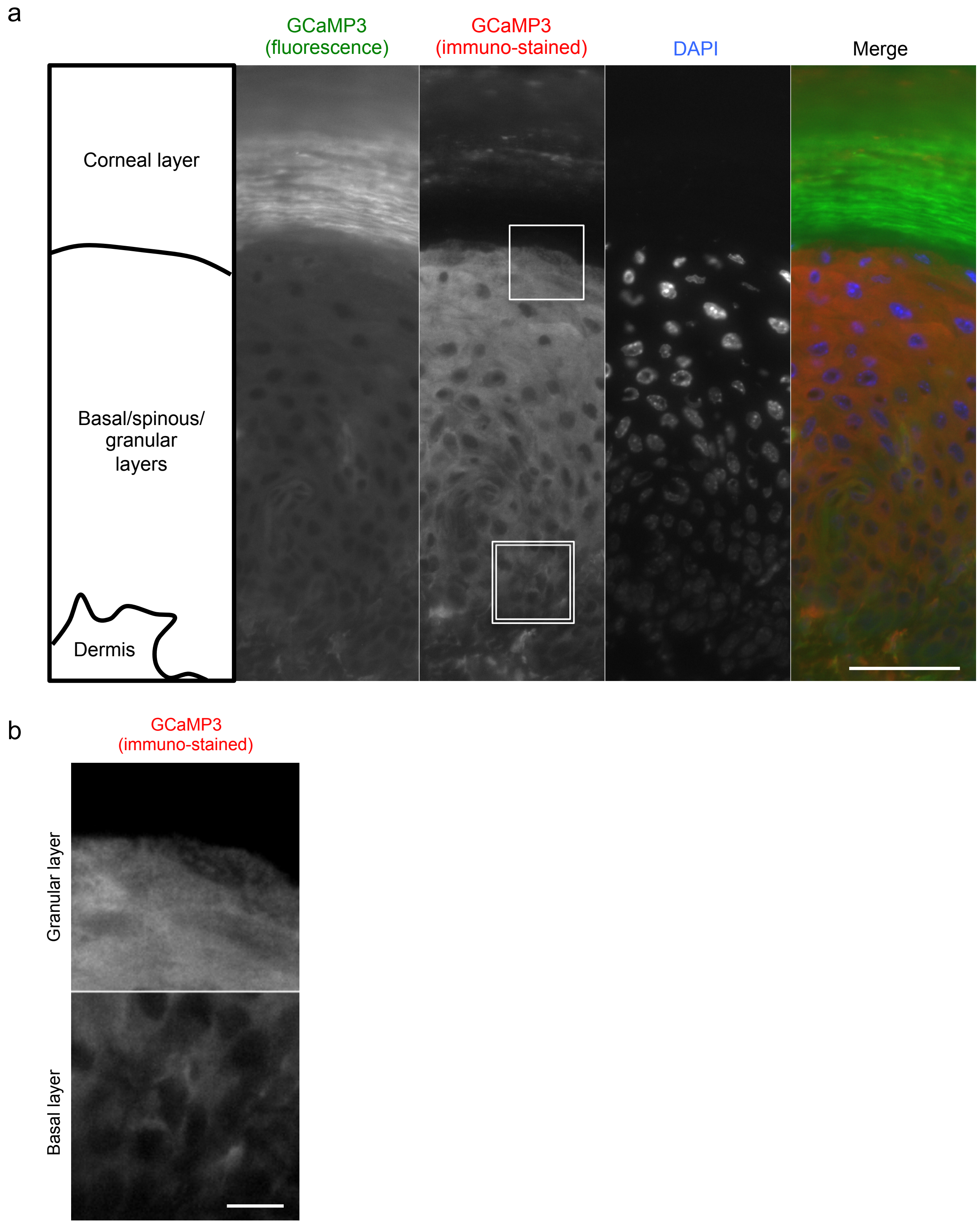

Supplemental Figure 1: Distribution of the protein of GCaMP3 in the different layers of the epidermis
(a) Low magnification view of a cryosection of the fingertip skin of GCaMP3 mice. The fluorescence of GCaMP3 was detected, but exhibited a monotonous intensity from the basal to granular layers; the contrast of fluorescence derived from high concentration of calcium ions was lost during the sample preparation. The protein of GCaMP3 was immunostained with anti-green fluorescent protein (GFP) antibody conjugated with Alexa-594. DNA was stained with DAPI. (b) High magnification view. Upper panel: granular layer at the square in (a). Lower panel: basal layer at the double-lined square in (a). Note that the contrast of distribution of the protein of GCaMP3 was lost in the granular layer. Scale bars: 50 µm (a) and 10 µm (b).

Supplemental Figure 2

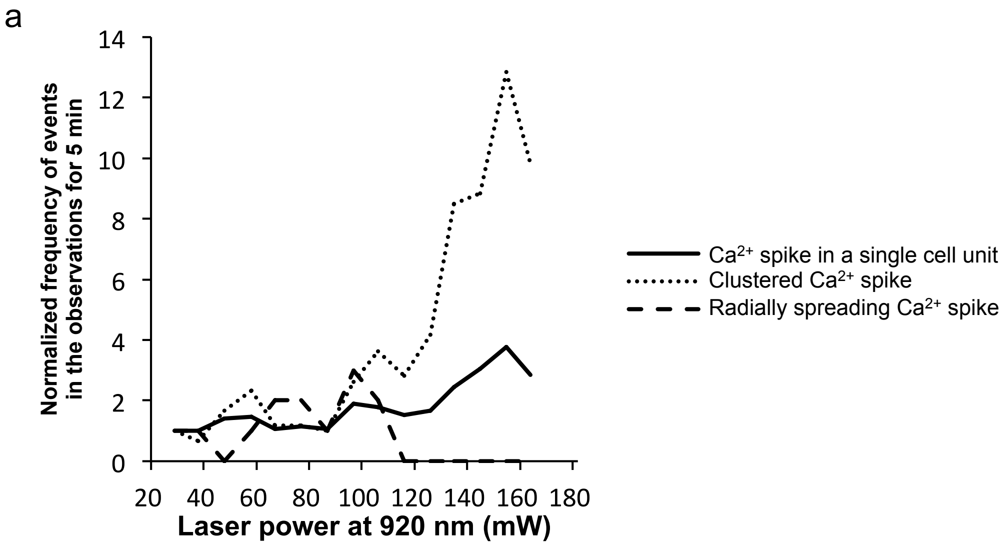

Supplemental Figure 2: Correlations between the intensity of excitation laser and the frequency of Ca^{2+} spikes in the basal layer
(a) Relationship between the relative frequency of Ca^{2+} spikes in the basal layer and the power of excitation laser in the ear of GCaMP3 mice. Time-lapse images were obtained for 5 min at an interval of 6 s at each intensity of excitation laser at 920 nm. The result is representative of two independent experiments.

Supplemental Figure 3

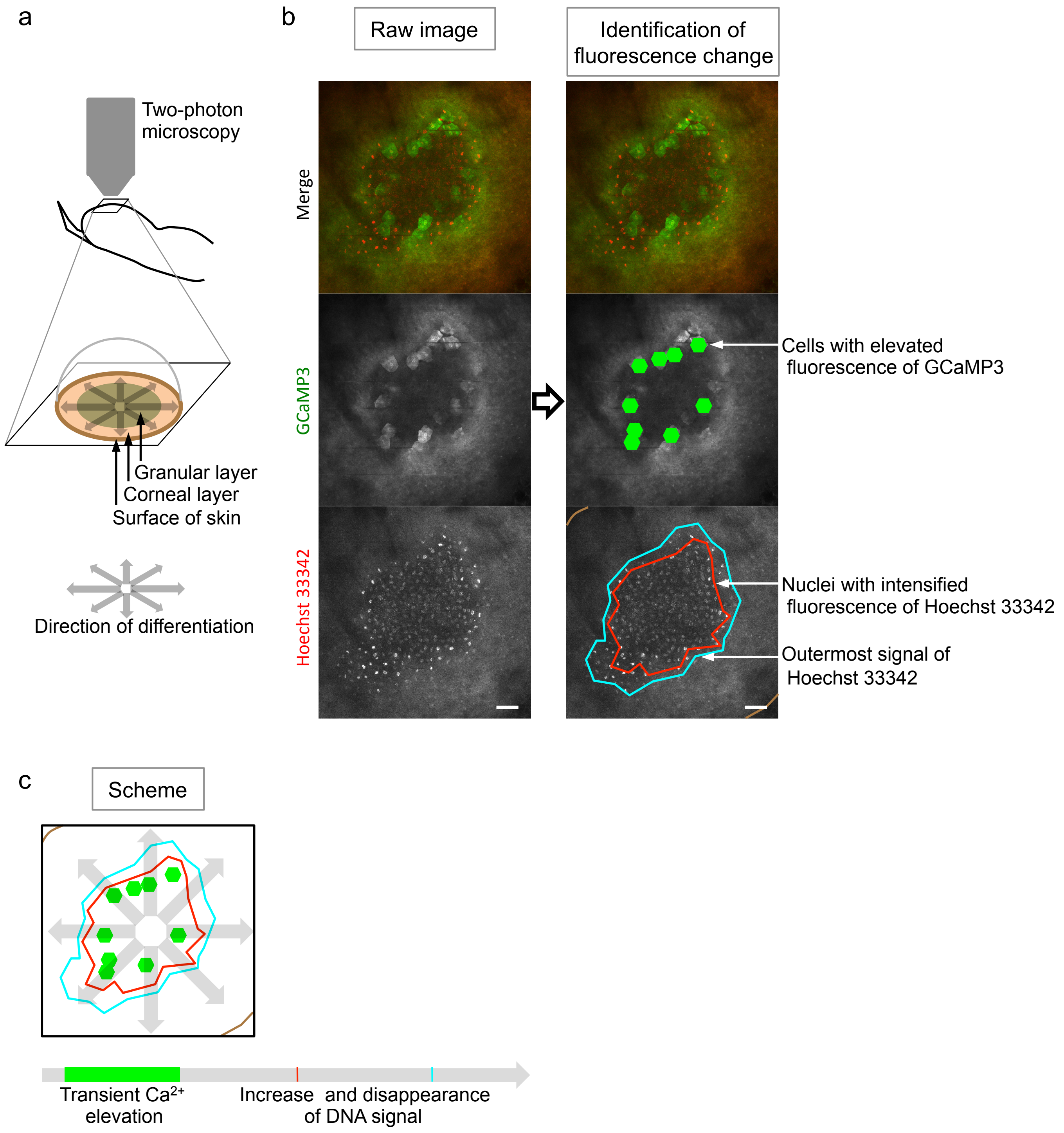

Supplemental Figure 3: Temporal changes in the DNA signal following the transient $[Ca^{2+}]_i$ elevation
(a) Schematic of imaging setup. (b) Representative two-photon image of the fingertip skin of mice systemically expressing GCaMP3, injected with Hoechst 33342 to label the DNA. Cells with an elevated fluorescence of GCaMP3 are marked with green hexagons. Nuclei with an intensified fluorescence of Hoechst 33342 are marked with a red line. The outermost signal of Hoechst 33342 is marked with a cyan line. The surface of the skin is marked with brown lines. (c) Schematic of the temporal changes in $[Ca^{2+}]_i$ and the DNA signal. Scale bar: 50 μm.

Supplemental Table: Settings for the observations by two-photon imaging

Devices

	Manufacturer	Name
Microscopy	Olympus (Tokyo, Japan)	IX-81
Objective lens	Olympus	UPlanSApo 30xSIR (NA: 1.05)
Excitation laser	Spectra-Physics (Santa Clara, CA)	Mai Tai Deep See

Stimulation and observation settings

Fluorophores	Wavelength (excitation, nm)	Wavelength (detection, nm)	Laser power (mW)
GCaMP3	920	495-540	38.8
ZO1-EGFP	920	495-540	38.8
Tandem dimer Tomato	920	575-630	58.2
mKO2-hCdt1 (FUCCI)	760	575-630	24.5
Hoechst 33342	760	420-460	136.5
Second-harmonic generation (collagen)	920	420-460	38.8

Frame rates

Basal layer	2-10 s
Granular layer	5-20 min
